# Foveal processing difficulty does not affect parafoveal preprocessing in young readers

**DOI:** 10.1038/srep41602

**Published:** 2017-01-31

**Authors:** Christina Marx, Stefan Hawelka, Sarah Schuster, Florian Hutzler

**Affiliations:** 1 Centre for Cognitive Neuroscience, University of Salzburg, Hellbrunnerstr. 34, 5020 Salzburg, Austria

## Abstract

Recent evidence suggested that parafoveal preprocessing develops early during reading acquisition, that is, young readers profit from valid parafoveal information and exhibit a resultant preview benefit. For young readers, however, it is unknown whether the processing demands of the currently fixated word modulate the extent to which the upcoming word is parafoveally preprocessed – as it has been postulated (for adult readers) by the foveal load hypothesis. The present study used the novel incremental boundary technique to assess whether 4^th^ and 6^th^ Graders exhibit an effect of foveal load. Furthermore, we attempted to distinguish the foveal load effect from the spillover effect. These effects are hard to differentiate with respect to the expected pattern of results, but are conceptually different. The foveal load effect is supposed to reflect modulations of the extent of parafoveal preprocessing, whereas the spillover effect reflects the ongoing processing of the previous word whilst the reader’s fixation is already on the next word. The findings revealed that the young readers did not exhibit an effect of foveal load, but a substantial spillover effect. The implications for previous studies with adult readers and for models of eye movement control in reading are discussed.

A key aspect of fluent reading is parafoveal preprocessing of upcoming words. It speeds up foveal word recognition and therefore leads to faster reading. As yet, most evidence on parafoveal preprocessing is based on studies with mature (i.e., adult) readers^1^. Evidence concerning the development of parafoveal preprocessing in beginning readers is comparatively scarce. Recent studies revealed that beginning readers soon begin to utilize information from parafoveal words for subsequent foveal word recognition, that is, they engage in parafoveal preprocessing and exhibit the resultant preview benefit. To illustrate, studies – using the *moving window paradigm*^2^ – showed that the perceptual span of 2^nd^ Graders (i.e., children with about one year of formal reading instruction) encompasses up to 11 letters to the right of fixation^3^,^4^, that is, the span corresponds to the length of two short words. Likewise, studies – using the *invisible boundary paradigm*^5^ – revealed that children in Grade 2 benefit from the availability of valid parafoveal information^6^,^7^. A study from our workgroup – using the novel *incremental boundary paradigm* – confirmed that 2^nd^ Graders benefit from valid parafoveal previews^8^. The extent to which the children were capable of parafoveal preprocessing, however, depended on the reading proficiency of the children, that is, more fluent readers exhibited a more pronounced preview benefit. The present study pursues the research on parafoveal preprocessing in children by examining whether children exhibit an effect of *foveal load*. Moreover, we attempted to distinguish the foveal load effect from the *spillover* effect.

The ease (or difficulty) with which we can process the currently fixated word can influence the processing times of neighbouring words^9^. The influence can – in principle – take two directions: On the one hand, the processing demands of the currently fixated word may modulate the extent to which the upcoming word is preprocessed – known as the effect of *foveal load*^10^. On the other hand, the processing of a word may not be completed when the next word is fixated, and completing the processing of the former word may affect fixation times of the next word – termed *spillover effect* (also known as *lag effect*^9^). Concerning the spillover effect, an early study of Rayner and Duffy^11^ reported inflated first fixation durations on a target word (henceforth word_n_) succeeding an infrequent pretarget word (henceforth word_n−1_). More recently, Kliegl and colleagues^9^ likewise showed that word frequency (as well as word length and predictability) of word_n−1_ affects fixation durations on word_n_ and discussed the possibility that this finding reflects spillover effects (see also^12^). However, these studies did not experimentally manipulate the preview of the upcoming words (e.g., by masking; see later). Thus, it is not clear whether these asserted spillover effects indeed reflect the completion of the processing of word_n−1_ or whether the findings reflect reduced parafoveal preprocessing of word_n_, that is, an effect of foveal load. Kliegl and colleagues^9^ acknowledged this fact by affirming that “*lag effects are compatible with explanations in terms of spillover (i.e., incomplete processing) and in terms of dynamic modulation of the perceptual span by foveal load” (p*. 28).

The foveal load hypothesis originated from a study by Henderson and Ferreira^10^. They reported that the frequency of the currently fixated word modulates the amount to which the next word is preprocessed. To be specific, the authors made use of the invisible boundary paradigm^5^. With this gaze-contingent paradigm it is possible to present an experimentally manipulated parafoveal preview (e.g., a different-letter mask) which is replaced by the actual word contingent on crossing an invisible boundary (between word_n−1_ and word_n_). Henderson and Ferreira^10^ applied three preview conditions: (i) valid previews (preview: *despite;* target: *despite*), (ii) visually similar previews (*desqlda*) and (iii) visually dissimilar previews (*zqdloyv*). The rationale of this manipulation is that only the preprocessing of valid (and visually similar) previews can result in a preview benefit. The foveal word was either a high-frequency or a low-frequency word (i.e., this was the manipulation of *foveal load*). The findings indicated a reduction of the preview benefit (in the valid and visually similar preview condition compared to the dissimilar previews) when word_n_ was preceded by a low-frequency word compared to instances in which word_n_ was preceded by a high-frequency word.

Critically, whether word_n−1_ was of high-frequency or of low-frequency did *not* affect processing times on word_n_ when parafoveal previews were visually dissimilar. This finding is of theoretical relevance, because – in case of a spillover effect – the “carried-over” processing of word_n−1_ would have influenced fixation times independent of the type of parafoveal preview. Put differently, this indicates that their finding for valid previews may indeed reflect an effect of foveal load and was not the result of a spillover effect (but see Discussion). Thus, it is the particular interaction of the frequency of word_n−1_ and the type of preview of the parafoveal word which supports the foveal load hypothesis. However, Warren and colleagues^13^ brought up a critical notion regarding the suitability of the dissimilar previews in order to assess the foveal load hypothesis. They argued that “*Henderson and Ferreira’s finding of an interaction may have been related to the interference caused by, and the reprocessing necessitated by, initially processing a nonsense string in the dissimilar preview condition*” (*p*. 8). Indeed, recent studies corroborated this critical assertion by showing that parafoveal masking inflicts processing costs (i.e., interference) for foveal word recognition in adult readers^14^,^15^ and in children^16^. In the present study, we did not utilize parafoveal masks, but used a visual degradation of the parafoveal preview of the word_n_ (i.e., we applied the *incremental boundary technique*^16^; see later).

As yet, the effect of foveal load has only been investigated in adult readers (and the evidence is mixed; see Discussion). Moreover, only one study attempted to distinguish the effect of foveal load from the spillover effect^17^ and – to our knowledge – no study attempted to do so in young, developing readers. In order to distinguish the spillover effect from the effect of foveal load we applied a novel technique, that is, the *incremental boundary paradigm*^16^. This paradigm combines the classical invisible boundary technique from the research field of eye movement control in reading^5^ with the rationale of the *incremental priming technique* from the research field of visual word recognition^18^. Figure 1 illustrates this combination. As aforementioned, with the invisible boundary technique one can manipulate the preview of the upcoming word in order to assess the magnitude of the preview benefit. The rationale of the incremental priming technique is to experimentally manipulate the perceptibility (i.e., the *salience*) of “primes”. If the presentation of a low-salience prime results in slower processing times of the target word, then it can be concluded that the (fully salient) prime is beneficial for processing (see ref. 18 for the logic of this *within-condition baseline*). In the present study, we manipulated the salience of the preview of word_n_ by displacing 0, 12 and 24% of its black pixels. Henceforth we refer to these three levels as high, medium and low salience, respectively. The degraded previews were replaced by the unmutilated word_n_ (and the post-target words) contingent on crossing an invisible boundary located immediately after the last letter of word_n−1_.

In a previous study^16^, we provided the proof-of-concept that visually degrading the parafoveal preview provides a (more) accurate measure of the preview benefit than the application of parafoveal masks. This advantage of the incremental boundary technique is indicated by the finding that the processing times of the target words converged for the valid preview condition and the masked preview condition in case the salience of the preview was low (i.e., when the preview was sufficiently degraded). This convergence of processing times (indexed by first fixation and gaze duration) indicates that the visually degraded previews neither facilitated nor interfered with processing of the target words.

The upper panel of Fig. 1 shows that the design of the present experiment was FREQUENCY OF WORD_N−1_ (high versus low frequency) by the SALIENCE OF THE PARAFOVEAL PREVIEW OF WORD_N_ (high, medium and low salience). The word frequency values for word_n−1_ were obtained from a child adequate database (i.e., the *childLex* database^19^). To recapitulate, the low-frequency words should induce high foveal load, whereas the high-frequency words should not induce such a load (which should be reflected by longer fixation times on the low-frequency than on the high-frequency words_n−1_). Reducing the salience of the parafoveal preview of word_n_ will impede the extraction of parafoveal information which should then be reflected in a reduction of the preview benefit^8^,^16^. Thus, the incremental boundary paradigm will allow us to relate the foveal load (induced by word_n−1_ frequency) and the different salience levels of the preview (word_n_ salience) to the ensuing processing time of word_n_ when it is eventually fixated (and presented normally, i.e., with full salience – contingent on crossing the invisible boundary). As explained next and illustrated in the lower panels of Fig. 1, this combination of experimental techniques is suitable for distinguishing between the effect of foveal load and the spillover effect.

The lower panels of Fig. 1 show hypothetical (and idealized) data. The left section depicts the pattern reflecting a spillover effect and the right section shows the pattern reflecting an effect of foveal load. In case we observe a spillover effect, children may preprocess the upcoming word to a comparable extent in both the high-frequency word_n−1_ and the low-frequency word_n−1_ condition. To be specific, word_n_ with high-salience previews will be processed faster than word_n_ with low-salience previews and this difference in processing speed indicates the extent of parafoveal preprocessing. However, the elevated extent of continuing processing a low-frequency word_n−1_ will result in a (generally) delayed recognition of word_n_. As a consequence, the lines in Fig. 1 are vertically shifted but remain parallel.

In case we observe an effect of foveal load, we expect – for the high-frequency word_n−1_ condition – that the children will exhibit a preview benefit on word_n_ due to (unconfined) parafoveal preprocessing. For the low-frequency word_n−1_ condition, in contrast, we expect that the processing time of word_n_ is not modulated by the different levels of salience – indicating that parafoveal preprocessing has not occurred (or – at least – it has been attenuated). In statistical terms: We expect a main effect of the frequency of word_n−1_ for both hypotheses, but – critically – in the case of a foveal load effect we expect an interaction of word_n−1_ frequency with the salience of the parafoveal preview (as in the original study of Henderson and Ferreira^10^). In case we observe a spillover effect we would expect no such interaction.

We investigated the hypothesized effects – spillover versus foveal load – in large samples of young readers from Grade 4 (*n* = 99) and Grade 6 (*n* = 139) with age-adequate reading skills. The experimental task required silently reading sentences which contained either a high-frequent or a low-frequent adjective as word_n−1_. Word_n_ was always a high-frequency noun (according to the *childLex* database^19^). For the statistical analyses, we administered linear mixed effects models (LMM). Fixed effects and group differences with a divergence of at least 2 standard errors (i.e., *t* > 2) were considered as significant (see Method section for details). To recapitulate, an effect of foveal load would be reflected by a significant interaction of the frequency of word_n−1_ and the salience of the parafoveal preview of word_n_. A (pure) spillover effect would be reflected in a main effect of word_n−1_ frequency in the absence of a significant interaction of word_n−1_ frequency with the salience of the parafoveal preview of word_n_. The choice of visual degradation of the parafoveal preview of the word_n_ (instead of parafoveal masking) will allow us to unambiguously interpret the effects of parafoveal preprocessing, because this salience manipulation is not prone to a misestimation of the preview benefit^16^ which would probably be an issue, if we used parafoveal masks^13^.

## Results and Discussion

### Global eye movement measures

As expected, the children from Grade 6 made fewer fixations per word than the children from Grade 4; *M* = 1.2 (*SD* = 0.16) and *M* = 1.4 (*SD* = 0.23), respectively; group difference: *b* = −0.172, *SE* = 0.039, *t* = −4.40. Likewise, the 6^th^ Graders exhibited shorter fixation durations than the 4^th^ Graders; *M* = 276 ms (40) and 308 ms (80); *b* = −0.156, *SE* = 0.018, *t* = −8.47. The mean forward saccade length of the 6^th^ Graders was longer than that of the 4^th^ Graders; *M* = 5.4 (1.0) and 4.9 (1.2); *b* = 0.463, *SE* = 0.134, *t* = 3.45. In correspondence with earlier studies from our lab^8^,^16^, the children of both Grades exhibited a similar proportion of regressions (*M* = 22% and 20% in Grade 4 and 6, respectively; *SD*s = 11 for both Grades; *b* = −0.021, *SE* = 0.015, *t* = −1.38).

### Pretarget words (word_n−1_)

As evident from Fig. 2 and in line with the original study of Henderson and Ferreira^10^ and other pertinent studies^17^,^20^,^21^, first fixation durations (FFD) and gaze durations (GD) were longer on the low-frequency word_n−1_ than on the high-frequency word_n−1_ (*b* = 0.290, *SE* = 0.032, *t* = 9.13 and *b* = 0.536, *SE* = 0.055, *t* = 9.71; respectively). Moreover, the LMM revealed significant two-way-interactions between word_n−1_ frequency and Grade (*b* = −0.071, *SE* = 0.026, *t* = −2.78 and *b* = −0.142, *SE* = 0.036, *t* = −3.97, for FFD and GD, respectively). Separate LMMs revealed that the fixed effects of frequency were larger for the 4^th^ Graders (FFD: *b* = 0.291; GD: *b* = 0.538) than for the 6^th^ Graders (FFD: *b* = 0.218; GD: *b* = 0.393). These analyses testify that our frequency manipulation of word_n−1_ revealed the expected effect.

### Target words (word_n_)

Figure 3 shows how FFD and GD on the word_n_ related to the preview of the word and to the frequency of word_n−1_. Table 1 provides the corresponding LMM results. As anticipated, the 6^th^ Graders exhibited shorter FFD and GD than the 4^th^ Graders (reflected by significant main effects of Grade). It is further evident from the Figure, that the children of both Grades exhibited substantially shorter FFD and GD with an increasing salience of the parafoveal preview of word_n_, that is, they exhibited a preview benefit (reflected by the main effects of salience; see Table 1). This finding replicates previous findings of our group^8^,^16^ and shows that visual degradation is an effective method for manipulating the extent to which information can be extracted from the parafoveal word.

**Figure 1:**
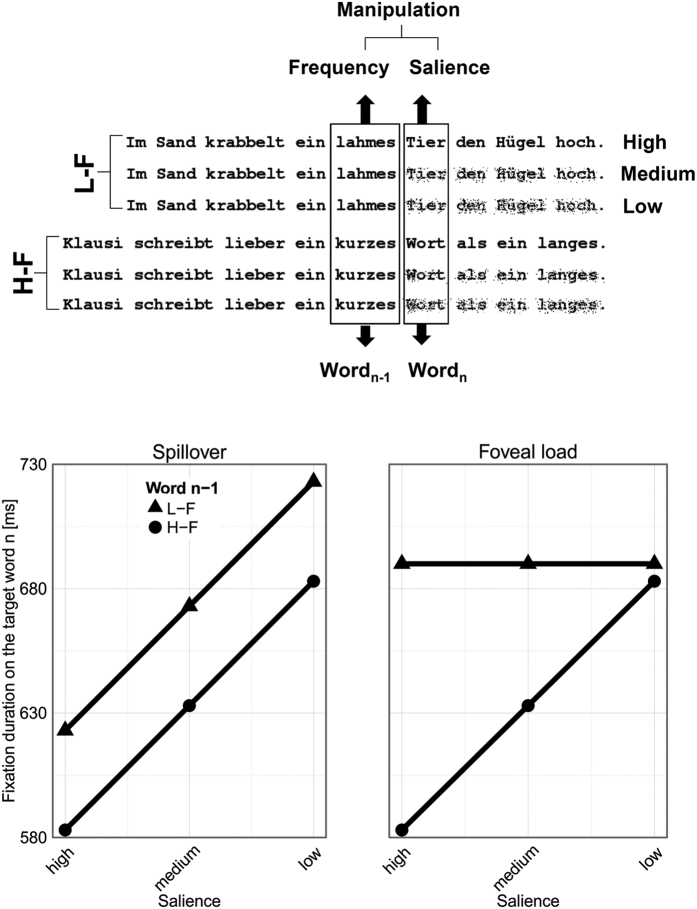
The upper panel shows two examples of the sentences of the experiment and illustrates the experimental manipulations. First, the sentences contained either a low-frequency word_n−1_ [L-F; lahm (*lame*)] or a high-frequency word_n−1_ [H-F; kurz (*short*)]. Furthermore, the figure illustrates our manipulation of the salience of the parafoveal preview of the word_n_. The lower panel schematically depicts the expected outcomes derived from the spillover and the foveal load account of parafoveal preprocessing. We expect a main effect of the frequency manipulation for both hypothetical scenarios, but only for the foveal load account we expect an interaction of word_n−1_ frequency with the salience of the parafoveal preview of the word_n_ (see main text for details). The scale of the y-axis is estimated on the basis of our previous study^16^ with children from Grade 4 and 6.

**Figure 2:**
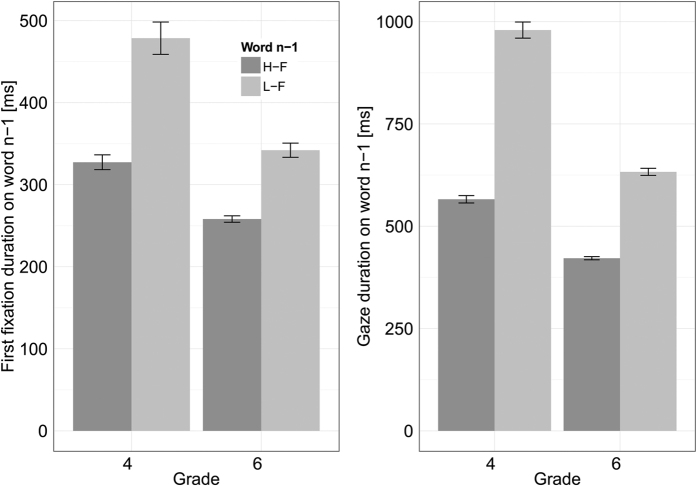
Mean first fixation and gaze duration on the word_n−1_ of 4^th^ and 6^th^ Graders. The dark grey bars show the mean duration on low-frequency word_n−1_ (L-F); the light grey bars show the mean duration on high-frequency word_n−1_ (H-F). Error bars represent one standard error of the mean (SEM).

**Figure 3:**
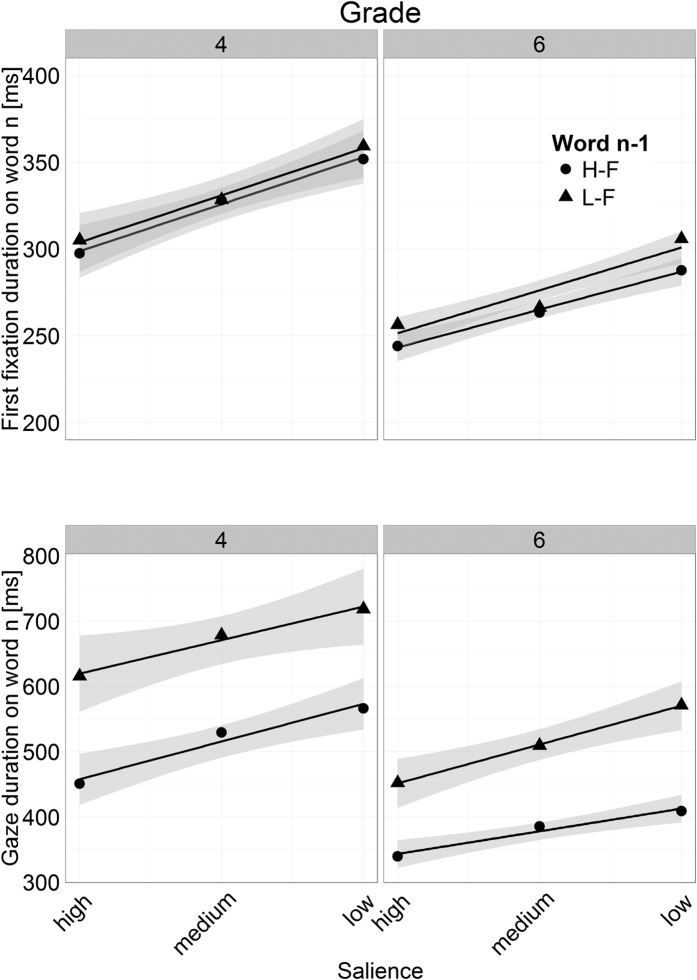
Mean first fixation and gaze duration on the word_n_ in relation to the salience of its parafoveal preview. The triangles display fixation times on word_n_ which were preceded by a low-frequency word_n−1_ (L-F); the circles refer to word_n_ preceded by a high-frequency word_n−1_ (H-F). The lines show the linear trends of fixation durations in relation to salience. The gray shadings depict 95% confidence intervals.

Figure 3 and Table 1 further reveal that the frequency manipulation of word_n−1_ did not elicit an effect on the FFD on word_n_. For GD, however, low-frequency of word_n−1_ resulted in prolonged durations on word_n_ for both Grades. Accordingly, for GD the LMM revealed a main effect of word_n−1_ frequency. Critically, (as evident by the virtual parallelism of the lines in Fig. 3) the two-way interactions between word_n−1_ frequency and the salience of the parafoveal preview of word_n_ were not significant – neither for GD nor for FFD. Likewise, all other interactions – including the three-way interaction between word_n−1_ frequency, word_n_ salience and Grade – were insignificant (see Table 1). To recapitulate, the parallelism of the lines indicates that word_n_ has been parafoveally preprocessed to a comparable amount – independent of the foveal load manipulation (i.e., word_n−1_ frequency; compare with the hypothetical results in Fig. 1). Thus, our findings clearly demonstrate that young readers exhibit a spillover effect of word_n−1_ frequency, but do not show a modulation of the extent of parafoveal preprocessing by foveal load.

A closer look at the replications of Henderson and Ferreira’s original study^10^ reveals that the effect of foveal load is modulated by several factors (in mature, adult readers). White and colleagues^20^, for example, showed that the awareness of the display change is a relevant factor. Their experimental setup closely resembled that of Henderson and Ferreira^10^ – relying on the invisible boundary paradigm and parafoveal masking. The authors compared the effect of foveal load of readers who did not consciously perceive the display change (i.e., the visual change from a parafoveal mask to the valid word) with those who did perceive the change. White and colleagues^20^ found that only readers who did not perceive the change showed an effect of foveal load. No effect of foveal load was found for readers who were aware of the display change. For these readers, the size of the preview benefit on word_n_ was unaffected by the frequency of the word_n−1_ – as was the case for the young readers of the present study. Concerning the present study, however, we doubt that awareness of the display change is accountable for the fact that we did not observe an effect of foveal load. White and colleagues^20^ purposefully chose dissimilar letter strings as parafoveal previews (e.g., word_n_: *girl*; preview: *bstc*) in order to induce awareness of the display change. Our visual degradation of parafoveal previews, by contrast, was comparatively subtle (i.e., instead of a massive change of letter identities it consisted only of a slight visual perturbation due to the replacement of a certain amount of pixels). Indeed, only two (out of the 238 participating children) reported that they perceived display changes when they were asked after the experiment whether they noticed something unusual.

**Figure 4:**
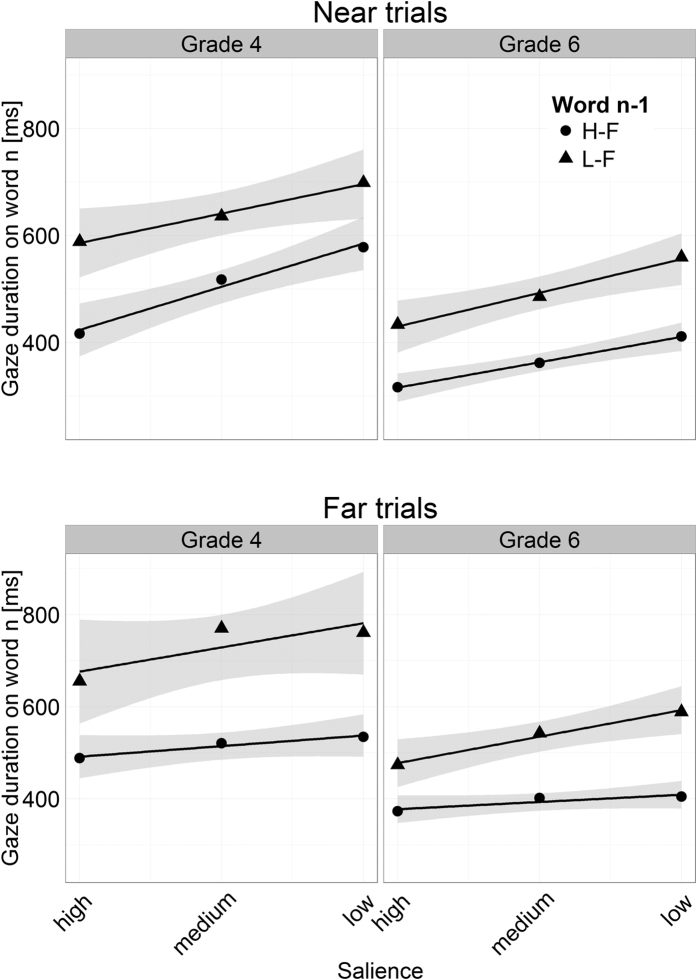
Mean gaze duration on word_n_ in relation to the salience of its parafoveal preview for the two launch-site distance classifications (i.e., near vs. far trials). Triangles display word_n_ preceded by a low-frequency word_n−1_ (L-F), circles refer to word_n_ preceded by a high-frequency word_n−1_ (H-F). The lines show the linear trends of gaze duration; the grey shadings depict 95% confidence intervals.

Another study^21^ reported that the effect of foveal load is modulated by the position of the last fixation prior to fixating word_n_ (i.e., the launch-site of the incoming saccade). In case where the last fixation was near to word_n_, the authors observed the predicted pattern postulated by the foveal load hypothesis. In the analysis, in which all trials (irrespective of launch-site distance) were considered, the authors did not find a differential effect of the frequency of word_n−1_ as predicted by the foveal load hypothesis (for first fixation duration, the effect was actually in the opposite direction, i.e., shorter fixations on word_n_ following a low-frequent word_n−1_). Motivated by the study of Kennison and Clifton^21^, we performed a re-analysis to assess whether our young readers exhibited an effect of foveal load when their last fixation before fixating word_n_ was near the word. To this end, we categorized the trials into “near trials” and “far trials”. As “near trials” we considered trials with launch-site distances of less than or equal to 3 characters from word_n_ (i.e., the last fixation was on the ultimate or penultimate letter of word_n−1_). As “far trials” we considered trials with launch-site distances greater than 3 characters. The results for GD (displayed in Fig. 4) do not show an effect of foveal load for the “near trials” – the critical interaction of word_n−1_ frequency with word_n_ salience was not significant (*b* = −0.017, *SE* = 0.028, *t* < 1; likewise, the analysis for FFD did not reveal such an interaction; *b* =  < 0.001, *SE* = 0.018, *t* < 1). Thus, unlike Kennison and Clifton^21^, we did not find an effect of foveal load even when the last fixation was close to word_n_.

**Figure 5:**
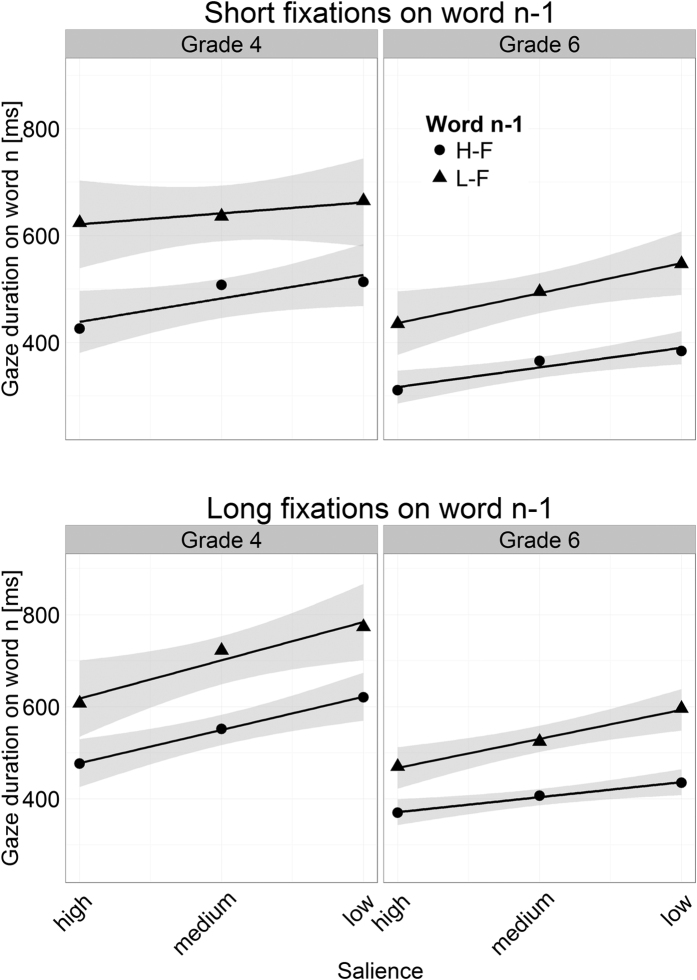
Mean gaze duration on the word_n_ in relation to the salience of its parafoveal preview separately for long and short (last) fixations on word_n–1_. Triangles display targets preceded by low-frequency word_n−1_ (L-F); circles refer to targets preceded by high-frequency word_n−1_ (H-F). The lines show the linear trends of gaze duration; the grey shadings depict 95% confidence intervals.

A particularly critical stance towards the foveal load hypothesis is conveyed by the work of Schroyens and colleagues^17^. They argued that the extent to which an upcoming word is preprocessed does not necessarily depend on the difficulty of the currently fixated word, but more on the fixation duration on the word (the distributions of fixation durations on high-frequency and low-frequency words overlapped considerably). It was suggested that parafoveal preprocessing is more pronounced in case of a long fixation and less pronounced in case of a short fixation. We performed a further re-analysis and categorized the trials on the basis of the duration of the last fixation on the word_n−1_. We categorized the trials into trials with “short” or “long” last fixations on word_n−1_ by median splitting the children’s fixation durations (the respective medians were 293 ms for the 4^th^ Graders and 239 ms for the 6^th^ Graders). As depicted for GD in Fig. 5, we found no interaction of the preview benefit with the fixation duration on word_n−1_, neither for the 4^th^ nor for the 6^th^ Graders (*t*s < 1 for both, GD and FFD). Thus, the absence of the foveal load effect for our young readers cannot be attributed to the variability in fixation durations on word_n−1_.

In essence, our main analysis did not reveal the effect predicted by the foveal load hypothesis and several auxiliary analyses showed that the absence of the effect cannot be attributed to several modulatory variables reported in the literature. The amount of parafoveal preprocessing (captured by contrasting high-salient with low-salient previews of word_n_) was not reduced when word_n−1_ induced a high foveal load (i.e., when it was low-frequent). Nonetheless, the frequency of word_n−1_ did substantially affect the processing times of word_n_. It did so, however, to the same extent for high-salient and low-salient previews. We observed substantially longer GD on word_n_ following a low-frequency word_n−1_ than on word_n_ following a high-frequency word_n−1_, that is, our young readers exhibited a substantial spillover effect.

Of interest is the specific pattern of the spillover effect of our young readers regarding our fixation duration measures. The spillover effect is assumed to affect FFD as well as GD (e.g. ref. 22). For our young readers, we found no spillover effect for FFD, but a substantial spillover effect for GD which is often considered as a “late measure” capturing the whole process of word recognition^23^ (i.e., up to lexical completion in terms of the E-Z reader model of eye movement control in reading^24^, and semantic integration). The evidence concerning the spillover effect for FFD is mixed. While some studies reported a spillover effect for FFD (e.g. ref. 22), other studies could not replicate this finding (e.g. ref. 17).

Recently, a study^25^ provided evidence that parafoveal preprocessing proceeds in two distinct phases. The first phase comprises an early orthography based “visual check” and is considered to be pre-attentional. The second phase is concerned with lexical access (and requires focused visual attention). Critically, the study showed that the first phase is not influenced by foveal load, whereas the later phase is. Thus, one possibility for the absence of a spillover effect in FFD is that young, developing readers may not (yet) proceed to the second phase of parafoveal preprocessing.

With regard to the late effect of word_n−1_ frequency (i.e., on GD), Balota and colleagues^22^ speculated that the spillover effect reflects the integration of words into the sentence context. In proficient, adult readers, contextual integration might already be reflected by early measures such as FFD. The less proficient, slower word recognition and the resultant delay of contextual integration in the young, developing readers of the present study could explain the “late” emergence of the spillover effect. However, the stimulus sentences were constructed to suit the reading skills of children and hence the low frequency pretarget words were probably not more difficult to integrate in the sentence context than the high frequency pretarget words. An alternative explanation for the late occurrence of the spillover effect could be that the “concepts” conveyed by a low-frequency adjective/noun pair is less “accessible” (for children) than a high frequency adjective/noun pair (note that the noun was always of high frequency). For a child it is probably easier to fathom the concepts of a “wild horse”, a “big ship” or a “young boy” than the concepts of a “lame animal” or the “sooty side [of the stove]”.

The absence of the foveal load effect and the finding of a spillover effect is of theoretical relevance with respect to models of eye movement control in reading. For the (serial-attention-shift) E-Z Reader model^24^, it is assumed that increasing processing difficulty of a word results in a decrement of the amount of time for preprocessing due to the late shifting of visual attention to the upcoming word. To be specific, in E-Z Reader only one word is processed at a time and visual attention is shifted towards the upcoming word when the processing of the currently fixated word is completed. Thus, the E-Z Reader model predicts an effect of foveal load, that is, it predicts prolonged fixation durations on word_n_, because foveal load (of word_n−1_) reduced the amount of preprocessing (of word_n_). The E-Z Reader does not, however, ascribe the prolonged fixation times to continued processing of word_n−1_ and thus does not predict a spillover effect.

The (guidance-by-attentional-gradient) SWIFT model, in contrast, can accommodate the finding of a spillover effect. The model assumes that several words (including the word left to the fixated words, i.e., word_n−1_) are processed simultaneously and that foveal load can lead to an inhibition of the execution of a saccade. Importantly, this inhibition due to foveal load may occur “time-delayed”, that is when the reader already fixates the word next to the word which actually induced the inhibition^26^. The purported reason for the “time-delay” is that the (cortical) word recognition process is slower than the autonomous saccade generation (in the brain stem)^27^,^28^.

We concede that the absence of a foveal load effect in the present study can be explained in several ways. It may be that young, developing readers do not exhibit an effect of foveal load (whereas mature, adult readers do). An assessment of this explanation would require (ideally) a cross-sectional (or longitudinal) study with a broader age range than that of the present study (to learn about the emergence of the foveal load effect during reading development). Another possibility could be that the previous findings of an ostensive effect of foveal load are due to the application of the classical invisible boundary technique. Recent evidence^14^,^15^,^16^ suggests that parafoveal masks (applied in conjunction with the classical approach) induce preview costs. As aforementioned, Warren and colleagues^13^ speculated that the application of parafoveal masks might lead to artificial interactions. Importantly, no preview costs and thus no artificial interactions are expected for the novel incremental boundary technique - providing a potential explanation for the absence of the effect of foveal load in the present study.

As aforementioned, there is evidence^25^ that parafoveal preprocessing proceeds in two stages. To reiterate, the first stage is concerned with coarse visual-orthographic preprocessing and the second stage is concerned with initiating lexical access. It could be that foveal load impedes (only) the second stage of preprocessing. Thus, it is possible that the critical interaction of load by preview in experiments on the effect of foveal load reflect an interfering effect of the dissimilar parafoveal preview in the low-load condition (were the preprocessing of the first stage discords with the preprocessing of the second stage), whereas no such an interference occurred in the high-load condition (where parafoveal preprocessing was confined to the first stage). The similar processing times for the target words after preprocessing a dissimilar preview in the two load conditions (as reported by Henderson & Ferreira), thus, may reflect the interference of the mask in the low-load condition and a spillover effect of word_n−1_ frequency in the high-load condition. That we refrained from using parafoveal masked but applied a salience manipulation for the parafoveal previews of word_n_ may avoided such an (artificial) interaction^16^,^29^. Of course, this interpretation is speculative and clarification would require a study which directly compares the application of parafoveal masks with the visual degradation of valid parafoveal previews.

### Conclusion and future direction

The current study is the first to investigate the effects of foveal load and spillover in young readers. The findings suggest no effect of foveal load in young readers. In fact, our 4^th^ and 6^th^ Graders exhibited a (late emerging) spillover effect of word_n−1_ frequency. For future studies, the incremental boundary technique might prove valuable to disentangle spillover and foveal load effects in adult readers.

## Method

### Participants

In total we tested 238 children from Grade 4 and Grade 6 (*n* = 99 and 139, respectively) from Primary Schools in the city of Salzburg and surrounding areas. Six children (2 from Grade 4, and 4 from Grade 6) were excluded from further analysis; one because of an autistic spectrum disorder, one because of a lack of proficiency in the German language, two because of poor performances on the comprehension questions of the experimental task (see below) and two because the calibration of the eye tracker did not succeed with these children. The remaining children (*n* = 232) had normal or corrected-to-normal vision and the children’s teacher reported no history of reading difficulties with these children. Finally, for assessing whether the children exhibited a normal reading speed (compared to respective age-norms) we conducted a screening of reading speed with a standardized test (*Salzburger-Lese-Screening SLS*^30^,^31^). In this test, children had to read lists of sentences which either formed semantically legal sentences conveying basic knowledge (e.g., “*A week has seven days*”) or semantically anomalous sentences which contradict basic knowledge (e.g., “*Strawberries are blue*”). Children had to mark each sentence as correct or incorrect within a time-limit of three minutes. Children with a below-average and above-average reading speed – defined as a reading quotient of less than 80 (*n* = 8; 2 from Grade 4) or more than 130 (*n* = 23, 16 from Grade 4) – were also excluded from further analyses. The final sample, therefore, consisted of 201 children: 79 4^th^ Graders (35 girls; 69 right handed; mean age: 10;0 *y*;*m, SD* = 0;5) and 122 6^th^ Graders (60 girls; 117 right handed; mean age: 11;9 *y*;*m, SD* = 0;5). The 4^th^ and 6^th^ Graders were comparable with respect to their age-related reading quotients of *M* = 106 and 104, respectively (*SD* = 10 in both Grades; *t*_199_ < 1.6).

Informed consent was obtained from all subjects and from one of their parents (or a legal guardian). The experiment was conducted in accordance with the Code of Ethics of the World Medical Association (Declaration of Helsinki) and was approved by the local ethics committee of the University of Salzburg (“Ethikkommission der Universität Salzburg”).

### Material

The children had to silently read 60 sentences in which we embedded one pretarget word (referred to as word_n−1_) and one target word (referred to as word_n_) per sentence. The experimental manipulation was twofold (see Fig. 1): Firstly, word_n−1_ was either a high-frequency (H-F) or a low-frequency (L-F) adjective with a mean length of 6.13 (*SD* = 0.63) and 6.43 (*SD* = 1.14), respectively (*t*_58_ = 1.27, *p* = .21). The H-F word_n−1_ had a mean frequency of their lemma form (i.e., the uninflected form of the word) of 1203 per million. The L-F word_n−1_ had a mean frequency of 29 per million (*t*_58_ = 4.63, *p* < .001). The word_n_ was always a high-frequency noun, with a mean length of 5 letters (range: 4 to 6 letters) and a mean frequency of 1160 (range: 169 to 4334) according to the *childLex* database for the age group 9 to 10-year olds^19^. Secondly, three preview conditions were generated for the parafoveal word_n_ by using the *pixmap-*package^32^ and an in-house *R-script*. In each preview condition all letters of word_n_ (and all words thereafter) were degraded, that is, a certain amount of black pixels were shifted. The amount of displaced pixels was 0, 12 and 24% for our three levels of degradation. Sentences were constructed in such a way that at least 4 words preceded and at least 1 word followed word_n_ (*M* = 5.3 and 3.2, respectively). The length of the sentences ranged from 7 to 13 words (*M* = 9.52, *SD* = 1.36). The sentences were typed in a bold and mono-spaced font type; black on white background. Each character had a width of 12 pixels on the display screen (whose specifications are provided in the apparatus section). From the 64 cm viewing distance a single character had a width of ~0.4° of visual angle.

### Apparatus

Eye movements were recorded for the right eye with a sampling rate of 500 Hz with a mobile EyeLink Plus (SR Research, Canada) using the “desktop mount” configuration with the “remote” setup which compensates for head movements (by tracking a target sticker on the child’s forehead). The experiment was presented on a 20 inch high-speed LCD-monitor (1024 × 768 pixel resolution with a 144 Hz frame rate).

### Procedure

First, we administered the reading speed test in the children’s classrooms. The eye movement experiment was conducted in a separate room (duration approx. 20 minutes). For the eye tracking task, we performed a horizontal 3-point calibration routine. The routine was repeated until the child achieved an average tracking error below 0.3° of visual angle. Then, five familiarization trials were administered. After every second familiarization trial a comprehension question was asked. Then, the calibration routine was repeated. Thereafter, we presented the 60 experimental sentences. A trial started with the presentation of a fixation cross on the left side of the screen (vertically centered). When the system detected a fixation on the fixation cross, the sentence was presented. The calibration routine was repeated when the fixation check failed and after each comprehension question (12 questions in total). Display changes were realized with the invisible boundary technique^5^. Children were instructed to silently read each sentence for comprehension. After each sentence, the child pressed a button on a Cedrus^®^ response box (RB-540, San Pedro, USA) to continue. Following 20% of the sentences, the children had to answer simple comprehension questions regarding the content of the preceding sentence. Inclusion criterion for further analysis was that the child answered at least 8 comprehension questions correctly. The mean correct answers were 11.62 and 11.69 for the 4^th^ Graders and the 6^th^ Graders, respectively. After the experiment, the experimenter asked the children whether they noticed “something unusual” about the presentation of the sentences.

### Data treatment and analyses

In total, we administered 12,060 trials (4,740 with the 4^th^ Graders; 7,320 with the 6^th^ Graders). After the removal of trials with data loss and outlying fixation times (i.e., fixations durations shorter than 80 ms) 9,432 and 9,459 trials remained for the analysis of first fixation duration (FFD) and gaze duration (GD), respectively. Eye movement data were analyzed by means of linear mixed effect models (LMM) using the *lmer*-function of the *lme4*-package^33^. For the global eye movement measures we considered each word except word_n−1_ (whose frequency was manipulated) and word_n_ (whose parafoveal preview was manipulated). The model assessed – as a fixed effect – the effect of Grade and accounted for the random effects of subjects (i.e., the individual children) and items (i.e., the individual words). The syntax for this model was *measure ~ grade* + (*1*|*subject*) + (*1*|*item*). For the analysis of processing times of the word_n−1_ we considered FFD and GD. The LMM assessed – as a fixed effect – the effect of Grade and word_n−1_ frequency and the two-way-interaction between these effects (again subjects and items were included as random effects: measure ~ grade * word_*n−1*_ *frequency* + (*1 | subject*) + (*1 | item*)). For the main analysis of the effect of word_n−1_ frequency and of the salience of the parafoveal preview of word_n_ on FFD and GD on word_n_, the syntax was as follows: measure ~ grade * word_*n−1*_ frequency * word_*n*_ *salience* + (*1 | subject*) + (*1 | item*). Further auxiliary models followed this principled scheme. Note that we *log*-transformed FFD and GD (by the natural logarithm) for the analyses. The 95% confidence intervals (95%), which are depicted in the Figures, were estimated with the *smooth*-function (method = “lm”) of the *ggplot*-package^34^.

## Additional Information

**How to cite this article**: Marx, C. *et al*. Foveal processing difficulty does not affect parafoveal preprocessing in young readers. *Sci. Rep.* **7**, 41602; doi: 10.1038/srep41602 (2017).

**Publisher's note:** Springer Nature remains neutral with regard to jurisdictional claims in published maps and institutional affiliations.

## Figures and Tables

**Table 1:**
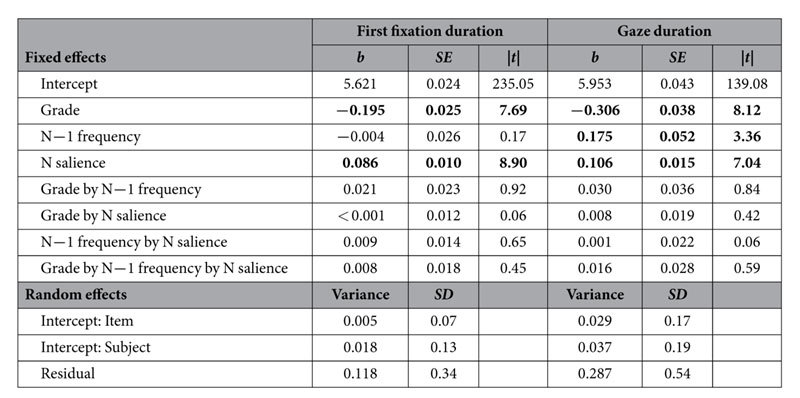
LMM estimates of fixed effects (upper part) and estimates of variance (lower part) for first fixation and gaze duration.

*Note:* Significant fixed effects a printed in bold.
